# Low-molecular-weight thiol transferases in redox regulation and antioxidant defence

**DOI:** 10.1016/j.redox.2024.103094

**Published:** 2024-02-24

**Authors:** Maria-Armineh Tossounian, Yuhan Zhao, Bess Yi Kun Yu, Samuel A. Markey, Oksana Malanchuk, Yuejia Zhu, Amanda Cain, Ivan Gout

**Affiliations:** a Department of Structural and Molecular Biology, University College London, London, WC1E 6BT, United Kingdom; b Department of Cell Signaling, Institute of Molecular Biology and Genetics, Kyiv, 143, Ukraine

**Keywords:** Antioxidants, Oxidative stress, LMW thiols, Thiol transferases, Defence mechanism

## Abstract

Low-molecular-weight (LMW) thiols are produced in all living cells in different forms and concentrations. Glutathione (GSH), coenzyme A (CoA), bacillithiol (BSH), mycothiol (MSH), ergothioneine (ET) and trypanothione T(SH)_2_ are the main LMW thiols in eukaryotes and prokaryotes. LMW thiols serve as electron donors for thiol-dependent enzymes in redox-mediated metabolic and signaling processes, protect cellular macromolecules from oxidative and xenobiotic stress, and participate in the reduction of oxidative modifications. The level and function of LMW thiols, their oxidized disulfides and mixed disulfide conjugates in cells and tissues is tightly controlled by dedicated oxidoreductases, such as peroxiredoxins, glutaredoxins, disulfide reductases and LMW thiol transferases.

This review provides the first summary of the current knowledge of structural and functional diversity of transferases for LMW thiols, including GSH, BSH, MSH and T(SH)_2_. Their role in maintaining redox homeostasis in single-cell and multicellular organisms is discussed, focusing in particular on the conjugation of specific thiols to exogenous and endogenous electrophiles, or oxidized protein substrates. Advances in the development of new research tools, analytical methodologies, and genetic models for the analysis of known LMW thiol transferases will expand our knowledge and understanding of their function in cell growth and survival under oxidative stress, nutrient deprivation, and during the detoxification of xenobiotics and harmful metabolites. The antioxidant function of CoA has been recently discovered and the breakthrough in defining the identity and functional characteristics of CoA *S*-transferase(s) is soon expected.

## Abbreviations

LMW: low-molecular-weight
GSH: glutathione
GSSG: glutathione disulfide
BSH: bacillithiol
BSSB: bacillithiol disulfide
MSH: mycothiol
MSSM: mycothiol disulfide
T(SH)_2_: trypanothione
CoA: coenzyme A
ET: ergothioneine
Cys: cysteine
GST: glutathione transferase
BST: bacillithiol transferase
MST: mycothiol transferase
TST: trypanothione transferase
Grx: glutaredoxin
GR: glutathione disulfide reductase
Gpx: glutathione peroxidase
Brx: bacilliredoxin
Bdr: bacillithiol disulfide reductase
Mrx: mycoredoxin
Mtr: mycothiol disulfide reductase
Prx: peroxiredoxin
H_2_O_2_: hydrogen peroxide
MAPEG: membrane-associated proteins in eicosanoid and glutathione metabolism
TCHQD: tetrachlorohydroquinine dehalogenase-like
DHAR: dehydroascorbate reductase
Fos: fosfomycin-resistance protein
VOC: vicinal oxygen chelate
MRP: multidrug resistance-related proteins
ABC transporters: ATP binding cassette transporters
keap1: Kelch-like ECH-associated protein 1
Nrf2: nuclear factor erythroid 2-related factor 2
JNK: Jun N-terminal kinase
PGH2: prostaglandin H2
PGD2: prostaglandin D2
oxPTM: oxidative post-translational modification
Met: methionine
Msr: methionine sulfoxide reductase
HOCl: hypochlorite
STL: S-transferase like
CDNB: 1-chloro-2,4-dinitrobenzene
BSRs: bacillithiol-electrophile conjugates

## 1 Introduction

**Fig. 1:**
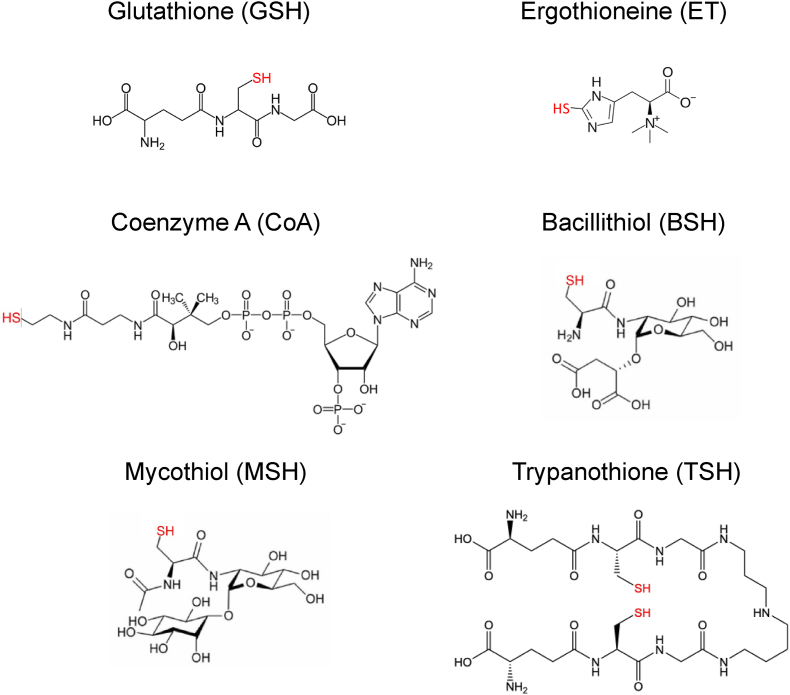
Structure of the main low-molecular weight (LMW) thiols. The structures of glutathione (GSH), ergothioneine (ET), coenzyme A (CoA), bacillithiol (BSH), mycothiol (MSH) and trypanothione (T(SH)_2_) are shown. The thiol (-SH) groups are indicated in red. The molecular weight (M_W_), pKa and redox potential of each LMW thiol are shown in Table 1. (For interpretation of the references to color in this figure legend, the reader is referred to the Web version of this article.)

Low-molecular-weight (LMW) thiols form a structurally diverse class of molecules with a functional and highly reactive sulfhydryl group. LMW thiols are found in all three domains of life, where they exhibit different expression profiles and play divergent functional roles. LMW thiols include glutathione (GSH), cysteine (Cys), coenzyme A (CoA), bacillithiol (BSH), mycothiol (MSH), trypanothione T(SH)_2_, ergothioneine (ET) and others (Fig. 1 and Table 1) [1]. The presence of a strong nucleophilic thiol moiety allows LMW thiols to be involved in diverse biochemical reactions of cellular metabolism, detoxification of reactive oxygen/nitrogen species and xenobiotics, maintenance of cellular redox homeostasis, as well as control of structural and functional properties of proteins through the formation and transfer of disulfide bonds [1,2]. Cellular functions of LMW thiols are facilitated via complex redox mechanisms involving dedicated antioxidant enzymes, such as disulfide reductases, peroxiredoxins, glutaredoxins, and LMW thiol transferases [[3], [4], [5], [6], [7]].

**Table 1:** Phylogenetic distribution and chemical properties of LMW thiols.

LMW thiol	Abbreviation	Phylogenetic distribution	Mw (Da)	Thiol pKa	Redox potential E°’ (mV)	LMW thiol transferase
**Glutathione**	GSH	Eukaryotes, most Gram-negative bacteria, few Gram-positive bacteria	307	8.93	−240	GST, FosA
**Bacillithiol**	BSH	Most low G + C Gram-positive bacteria, few Gram-negative bacteria	398	7.97	−221; −300 to −270	FosB, BstA
**Mycothiol**	MSH	Actinomycetes	486	8.76	−230; −300	MST
**Trypanothione**	T(SH)_2_	Trypanosomatids	722	7.4	−242	TST
**Ergothioneine**	EGT	Fungi and Actinomycetes	229	5.31	−60	Unknown
**Coenzyme A**	CoA	Ubiquitous	767	9.83	−234	Unknown

LMW thiol transferases constitute a family of multifunctional enzymes which play critical roles in cellular metabolic and detoxification processes. To date, thiol transferases for GSH, BSH, MSH and T(SH)_2_ have been identified and characterized [[8], [9], [10], [11], [12]]. A common feature of all LMW thiol transferases is their ability to catalyse the nucleophilic conjugation of specific thiols to a wide variety of exogenous and endogenous electrophiles, neutralizing their detrimental effects on cellular functions or modifying protein substrates (*S*-thiolation) under oxidative or metabolic stress. In addition, some LMW thiol transferases are known to possess peroxidase or isomerase activities and exhibit non-catalytic functions towards a wide range of endogenous and exogenous ligands [[13], [14], [15], [16]].

The superfamily of GSH transferases (GSTs) is the most extensively studied and widely distributed in all life forms [8]. The knowledge generated from the structure and function of GSTs has provided a basis for the identification and functional characterization of other LMW thiol transferases, including bacillithiol and mycothiol transferases. Initially, GSTs were the subject of intense investigations because of their ability to facilitate detoxification of herbicides and other toxic compounds. Numerous studies of mammals, insects, microbes, and plants have reported the involvement of GSTs in the conjugation of glutathione to a vast array of electrophilic entities, mediating the detoxification of xenobiotics. Subsequent studies revealed that some GSTs are also involved in protecting proteins (glutathionylation) from oxidative stress [17,18]. It is well known that surface exposed cysteine residues are susceptible, under oxidative stress, to reversible oxidation to sulfenic acid which is highly reactive and unstable. This modification primes the sulfenic acid to form intra/intermolecular disulfide bonds with nearby reduced cysteine residues or form a mixed disulfide bond with LMW thiols, termed protein thiolation. These disulfide bonds protect cysteines from overoxidation to sulfinic and sulfonic acids under prolonged oxidative stress. The latter modification is irreversible, targeting the protein for degradation. Under oxidative stress conditions, protein thiolation by GSH, BSH, MSH, T(SH)_2_ and CoA, has been reported [[19], [20], [21], [22], [23], [24]]. Protein thiolation is largely non-enzymatic, but the catalytic involvement of LMW thiol transferases (e.g. GSH transferases) has been reported.

LMW thiol transferases produced in eukaryotic and prokaryotic cells have gathered much attention due to their implication in neurodegenerative pathologies, detoxification of electrophilic toxins such as carcinogens, drug metabolism and antibiotic resistance.

## 2 GSH and glutathione transferases

### 2.1 GSH and its cellular functions

**Fig. 2:**
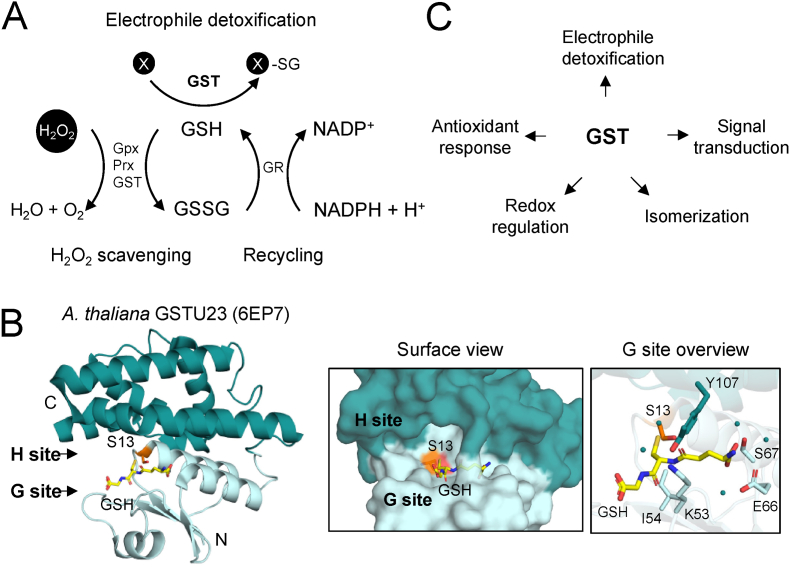
Structural architecture and cellular functions of glutathione transferases. **(A)** GSH participates in diverse cellular functions including electrophile (X) and H_2_O_2_ detoxification, which are catalyzed by GST and Gpx/Prx/GST, respectively. Following H_2_O_2_ scavenging, GSSG is released, and is reduced by the NADPH-dependent GR into two molecules of GSH. GSH also protects proteins from overoxidation through the formation of a mixed disulfide bond with protein thiols, termed protein glutathionylation (not shown). **(B)** The *A. thaliana* GSTU23 X-ray structure (PDB: 6EP7) is shown. The N-terminal Trx-like domain and the C-terminal helical bundle domain are shown in light and dark cyan, respectively. The glutathione (G site) and hydrophobic substrate (H site) binding sites are indicated with arrows. The left insert shows a surface view of the GSTU23 G and H sites. The right insert shows the residues (cyan) and water molecules (cyan circle) that stabilize GSH via H-bonds within the G site. The catalytic Ser13 is shown in orange sticks, and GSH in yellow sticks. The N-terminus (N) and C-terminus (C) are indicated. **(C)** GST participates in diverse cellular functions, including electrophile detoxification, signal transduction, antioxidant response, redox regulation of proteins and isomerization reactions. (For interpretation of the references to color in this figure legend, the reader is referred to the Web version of this article.)

Glutathione (GSH; l-γ-glutamyl-L-cysteinyl-glycine) is a key cellular antioxidant found in plants, animals, most Gram-negative bacteria and sporadically in Gram-positive bacteria [[25], [26], [27]]. It participates in diverse cellular functions, including antioxidant response, redox regulation, detoxification of xenobiotics, iron metabolism, signal transduction, and gene expression [[28], [29], [30], [31], [32], [33], [34], [35], [36]] (Fig. 2A). During cellular stress, GSH plays a major role as an antioxidant by protecting proteins from oxidative damage through the formation of a mixed disulfide bond with protein cysteines (PS-SG), termed protein glutathionylation. This modulates the activity, subcellular localization and regulatory interactions of modified proteins. Glutaredoxin (Grx) is an antioxidant enzyme that catalyzes the reduction of the mixed disulfide bond (PS-SG), resulting in the transfer of GSH from the protein onto Grx (GrxS-SG). Another molecule of GSH then reduces the second mixed disulfide bond (GrxS-SG), where glutathione disulfide (GSSG) is formed. Glutathione disulfide reductases (GR) reduce the GSSG into two molecules of GSH and use NADPH as the final electron donor [37,38]. Antioxidant enzymes, such as glutathione peroxidases (Gpx), peroxiredoxins (Prx), and some glutathione transferases (GST; formerly known as glutathione *S*-transferases) utilize GSH to reduce the pool of oxidants (e.g. hydrogen peroxide (H_2_O_2_)) generated during oxidative stress. In addition to its role as a redox buffer, GSH participates in scavenging of both endogenous and exogenous electrophiles, which are catalyzed by GSTs. The diverse cellular functions of GSTs and their role in redox regulation and signaling are discussed in the following sections.

### 2.2 Glutathione transferase superfamilies and classes

GSTs are evolutionarily conserved enzymes present ubiquitously in almost all forms of life, from bacteria to plants and animals. These enzymes were first discovered in animals due to their importance in metabolism and detoxification of drugs [[39], [40], [41]]. Three major GST superfamilies have been reported: the cytosolic GSTs, mitochondrial GSTs (also known as the Kappa class), and microsomal GSTs (known as membrane-associated proteins in eicosanoid and glutathione metabolism (MAPEG)). Cytosolic GSTs are the most abundant and are evolutionarily distinct from mitochondrial and microsomal GSTs [42,43]. Information on the different superfamilies and classes of human GSTs are summarized in Table 2.

**Table 2:** Human glutathione transferases. The table summarizes the superfamily, classes, and isoforms of human GSTs. The table also contains the molecular weight, UniProt accession number, protein oligomerization state, known structures of the GST isoforms and the tissue expression profile. GST expression levels vary in different tissues. Information within this table was gathered from UniProt and literature [44,45].

Superfamily	Class	Isoforms	UniProt (accession code)	Subunits	Amino acid number	Structure (PDB code)	Tissue expression
**Cytosolic**	**Alpha**	GSTA1	P08263	Homodimer; Heterodimer GSTA1-2	222	1GSD; 1PKZ	Testis, Liver, kidney, adrenal gland, pancreas
		GSTA2	P09210		222	1AGS; 2VCT	Liver, testis, pancreas, kidney
		GSTA3	Q16772	Homodimer	222	1TDI; 2VCV	Placenta
		GSTA4	O15217		222	1GUL; 1GUM	Small intestine, spleen, liver, kidney
		GSTA5	Q7RTV2	–	222	–	–
	**Zeta**	GSTZ1	O43708	Homodimer	216	1FW1	Fetal liver, skeletal muscle, kidney
	**Theta**	GSTT1	P30711	Homodimer	240	2C3N; 2C3Q	Kidney, liver, small intestine, brain
		GSTT2	P0CG30		244	1LJR; 4MPG	Liver, lung
	**Mu**	GSTM1	P09488	Homodimer	218	1GTU; 1XW6	Liver, testis, brain, adrenal gland, kidney, lung
		GSTM2	P28161		218	1HNB; 2C4J	Brain, skeletal muscle, testis, heart, kidney
		GSTM3	P21266		225	3GTU	Testis, brain, small intestine
		GSTM4	Q03013		218	4GTU	Brain, heart skeletal muscle
		GSTM5	P46439		218	–	Brain, heart, lung, testis
	**Pi**	GSTP1	P09211	Homodimer	210	10GS; 16GS	Liver, brain, heart, lung, testis, kidney, pancreas
	**Sigma**	GSTS (**HPGDS)**	O60760	Homodimer	199	1IYH; 2CVD	Adipose tissue, fetal liver, bone marrow
	**Omega**	GSTO1	P78417	Homodimer	241	1EEM; 5YVN	Liver, pancreas, skeletal muscle, spleen, heart
		GSTO2	Q9H4Y5	–	243	3Q18; 3Q19	Testis, liver, kidney, skeletal muscle, prostate
**Mitochondrial**	**Kappa**	GSTK1	Q9Y2Q3	Homodimer	226	1YZX; 3RPN	Liver, kidney, adrenal gland
**Microsomal**	**MAPEG**	MGST1	P10620	–	155	–	Liver, pancreas, prostate, colon, kidney, brain
		MGST1-like 1	O14684	Homotrimer	152	3DWW; 4AL0	Testis, prostate, small intestine, colon
		**MGST2**	Q99735		147	6SSR; 6SSS	Liver, skeletal muscle, small intestine, testis
		MGST3	O14880	–	152	–	Heart, skeletal muscle, adrenal gland, thyroid
		LTC4S	Q16873	Homotrimer	150	2UUH; 3PCV	Platelets, lung, liver
		FLAP	P20292		161	6VGC; 6VGI	Lung, spleen, thymus

GST superfamilies are divided into different classes, which share more than 40% amino acid sequence identity between isoforms of the same class [46,47]. The number of GST classes differ in different organisms, where Theta and Zeta class GSTs are found in animals, plants and bacteria, while other GST classes are more specific to animals (e.g. Alpha, Mu, Pi, Omega, Kappa, and Sigma classes) or plants (e.g. Phi, Tau, Lambda, tetrachlorohydroquinine dehalogenase-like (TCHQD) and dehydroascorbate reductase (DHAR) classes) [[46], [47], [48], [49], [50]]. Bacterial GST classes also include the Beta and Chi GSTs [46,51,52]. In some bacteria, the fosfomycin-resistance protein (FosA) from the vicinal oxygen chelate (VOC) superfamily is present. FosA is a metal-dependent GST that catalyzes the conjugation of GSH onto fosfomycin, an antibiotic. This conjugated product has no bactericidal properties [53]. Different GST classes can have distinct subcellular localization, function and structural features. For example, Phi and Tau classes are the most abundant plant GSTs that have a catalytic serine residue and possess GSH transferase and peroxidase activities [54,55]. On the other hand, Lambda and DHAR GST classes have a cysteine as their catalytic residue and catalyse GSH-dependent thioltransferase and dehydroascorbate reductase activities [43,56]. GSTs occupy different subcellular localizations (e.g. cytosol, mitochondria, microsome). Interestingly, GST Pi was found to translocate from the cytosol to mitochondria, the endoplasmic reticulum, and the nucleus [[57], [58], [59]]. This suggests that it could play an important role in these different subcellular compartments. A study analyzing the tissue distribution of GSTs showed that cytosolic GSTs are highly present in liver, kidney, adrenal glands and blood [60] (Table 2).

### 2.3 Structural architecture of glutathione transferases

Most cytosolic glutathione transferases are homodimeric enzymes and have a canonical fold composed of an N-terminal thioredoxin-like domain (domain 1) and a C-terminal helical bundle domain (domain 2) [54,55,61] (Fig. 2B). Domain 1 is highly conserved among the different GST classes and contains the thioredoxin fold which is composed of 4 β-sheets and 3 α-helices. A SNAIL/TRAIL motif has been reported in domain 1 of most GSTs [46,62,63], which includes residues that stabilize GSH within the active site. Domain 2 contains different number of α-helices depending on the type of the GST class and is responsible for binding to different electrophilic substrates [46]. Extra features have been observed in some GST classes, such as the “Mu loop” that makes the active site cleft of the Mu class GSTs deeper [15,63,64], and an extra α-helix in domain 2 of Alpha class GSTs that causes the formation of a smaller hydrophobic substrate binding site. The cytosolic and mitochondrial GST structures have similarities and differences [65]. An example is the structure of GST Kappa-1-1 that has an alternative structural arrangement of the two domains discussed, where the helical bundle domain is inserted within the core of the thioredoxin-like domain [65]. The cytosolic and mitochondrial GSTs do not share structural similarities with the MAPEG GSTs [66]. The homodimeric bacterial Beta class GSTs have a conserved canonical GST fold, as described for cytosolic GSTs; however, the dimer interface is polar, compared to the mostly hydrophobic dimer interface of cytosolic GSTs [53].

The GST active site is composed of a GSH binding site, known as the “G site”, and the electrophilic/hydrophobic substrate binding site, known as the “H site”. The G site is mainly composed of residues within domain 1, including the catalytic residues. GST classes have different types of catalytic residues, which include cysteine, serine, or tyrosine [37,46]. Upon binding of GSH to the G site, the catalytic residues (Cys, Ser, Tyr) lower the pKa of the GSH thiol from 8.93 to 6–7 and stabilize its thiolate anion form (GS^−^) through H-bonding [37,[67], [68], [69], [70]]. This facilitates the interaction between GS^−^ and the electrophilic/hydrophobic substrates. The X-ray crystal structure of homodimeric *Arabidopsis thaliana* (*A. thaliana*) GST Tau23 (GSTU23 – PDB: 6EP7) [54] is used as an example in this review to represent the common GST structural fold. GSTU23 has two domains (Fig. 2B), the N-terminal thioredoxin-like domain 1 (light cyan) and the C-terminal helical bundle domain 2 (dark cyan). GSH is located within the G site and its thiol group is at a H-bond distance from the catalytic Ser13. It mainly interacts with the G site residues, including the H-bonds formed between the GSH γ-glutamyl moiety and Glu66/Ser67, the GSH glycyl moiety and Lys40/Lys53, and the backbone of GSH Cys with the backbone of Ile54. In addition, GSH also interacts with other G site residues through water molecules [54].

### *2.4* Diverse cellular functions of glutathione transferases

Glutathione transferases are Phase II detoxification enzymes that catalyse the conjugation of GSH onto various electrophilic compounds (RX) [71]. The R and X stand for the type of electrophile (e.g. aromatic, heterocyclic or aliphatic compound) and leaving group (e.g. halide ions, sulfate, and nitrile) of the compound, respectively (Fig. 2C). The generated conjugates are mainly more soluble and are exported out of the cell by multidrug resistance-related proteins (MRP) [72]. MRP1 and MRP2 are ATP binding cassette (ABC) transporters that export GSH conjugated compounds [73,74]. This is recognized as the first of the four steps necessary for the mercapturic acid synthesis that leads to the elimination of harmful compounds from the cell [71]. Reported exogenous substrates of soluble GSTs include herbicides, pollutants, carcinogens, drugs, cancer chemotherapeutic agents and environmental chemicals [42,47,[75], [76], [77]].

Cytosolic GSTs participate in the cellular antioxidant response by metabolizing endogenous compounds (e.g. H_2_O_2_ and lipid peroxides) generated during cellular oxidative stress. Superoxide dismutases, catalases, glutathione peroxidases (Gpx), peroxiredoxins (Prx) and GSTs are antioxidant enzymes that scavenge these harmful oxidants and prevent the damage of cellular macromolecules. Some GSTs catalyse the glutathione-dependent reduction of H_2_O_2_ and lipid/organic peroxides into water and alcohol, respectively (Fig. 2A and C). GSSG is released at the end of the reaction. For example, *A. thaliana* GST Phi9 (GSTF9) can reduce H_2_O_2_, *tert*-butyl hydroperoxide, and cumene hydroperoxide [55], and the rat liver GST8-8 is reported to have hydroxyalkenals (e.g. 4-hydroxynon-2-enal) as substrates, which are end products of lipid peroxidation [78]. Although some GSTs possess the glutathione-dependent peroxidase activity, their rate constants have been reported to be significantly lower compared to Gpx and Prx [55,[79], [80], [81], [82], [83]].

In addition to the detoxification and GSH-dependent peroxidase activities, GST members have been reported with other functions (Fig. 2C). A study showed that GST Pi (GSTP1) can regulate peroxiredoxin 6 (PRDX6) through the conjugation of glutathione onto the protein [84]. In the presence of GSSG, GSTP1 is also reported to glutathionylate IKKβ (inhibitor of nuclear factor kappa-B kinase subunit β), thus activating NF-κB (Nuclear factor kappa-light-chain-enhancer of activated B cells), which is a key transcription factor that activates inflammatory genes [85]. Another study described the potential role of GSTP1 in catalyzing the glutathionylation of Kelch-like ECH-associated protein 1 (Keap1), that activates Nrf2 (nuclear factor erythroid 2-related factor 2) [86]. Nrf2 regulates the expression of hundreds of genes that encode proteins with antioxidant and anti-inflammatory functions. Omega class GSTs (GSTO1-1 and GSTO2-2) are reported to function as dehydroascorbate (DHA) reductases by catalyzing the reduction of DHA to ascorbic acid (AA), which is an important cellular antioxidant [[87], [88], [89]]. Specific types of GST classes participate in signal transduction, for example, Pi class GSTs were shown to regulate the function of Jun N-terminal kinase (JNK), and Alpha and Mu class GSTs were reported to regulate cell signaling by interacting with kinases [90,91]. Some GST class members participate in isomerization reactions including the Zeta class GSTs that play a role in tyrosine and phenylalanine catabolism by catalyzing the isomerization of maleylacetoacetate to fumarylacetoacetate in the presence of GSH [92]. Human GST Alpha3-3 possesses high steroid double bond isomerase function by catalyzing the conversion of Δ5-androstene-3,17-dione to Δ4-androstene-3,17-dione [93]. Among GST members are those that participate in hormone biosynthesis. Mammalian Sigma class GSTs (known as prostaglandin D synthase) can catalyse the isomerization of prostaglandin H2 (PGH2) to prostaglandin D2 (PGD2) in the presence of GSH [94].

Bacterial GST classes have been shown to participate in different cellular processes, including thiol disulfide oxidoreductase reactions, GSH conjugation and metabolic reactions (e.g. biotransformation of dichloromethane, and reductive dichlorination of pentachlorophenol) [53]. Overall, GSTs have diverse functions which regulate important cellular processes.

### 2.5 Redox regulation of GSTs and their role in stress response

During oxidative stress, cysteine and methionine residues are most susceptible to oxidation. Oxidative post-translational modifications (oxPTMs) on proteins include cysteine thiol oxidation (e.g. sulfenic acid, sulfinic acid, sulfonic acid, intra-/intermolecular disulfide bonds and mixed disulfide bonds) and methionine oxidation (e.g. methionine sulfoxide and methionine sulfone). Protein oxidation can alter the function, structure, and downstream interactions of proteins [[95], [96], [97], [98]]. Studies have reported the redox regulation of GSTs through oxPTMs [54,55,99]. Oxidation of methionine residues on GST Phi9 (GSTF9), GST Tau21 (GSTU21) and GST Tau23 (GSTU23) were identified by a proteomic study performed on stressed *A*. *thaliana* leaves [99]. Further biochemical studies showed an inhibition of both GSTF9 and GSTU23 GSH transferase activities upon methionine oxidation. The activities were restored in the presence of methionine sulfoxide reductases A (MsrA) and B (MsrB), which are antioxidant enzymes that reduce oxidized methionine residues [54,55]. Within both X-ray crystal structures, oxidized methionine residues were located mainly within the vicinity of the catalytic sites. Redox regulation of GSTs through cysteine oxidation is also reported. In the presence of H_2_O_2_, aside from methionine oxidation, GSTU23 is reported to form an intramolecular disulfide bond with cysteine residues located close to the active site. This results in a decrease in its catalytic activity, which is restored by the GSH/Grx/GR pathway [54]. In different studies, glutathione transferases were identified in the sulfenome (sulfenic acid containing proteins) and sulfinome (sulfinic acid containing proteins) of HeLa and A549 cells subjected to oxidative stress [100]. Diamide-stressed HEK293/Pank1β cell lysates analyzed by mass spectrometry showed CoAlation on GST Kappa1, GST Mu3, GSTO1, GSTP and microsomal GST1 and GST3 [101]. All these oxPTMs can regulate the diverse functions of GSTs during cellular oxidative stress.

An increase in GST expression is observed in bacteria, flies, fish, mammals and plants during cellular stress [[102], [103], [104], [105], [106], [107], [108]]. GST genes are regulated by Nrf2, which controls the expression of stress response genes during cellular stress [109]. Nrf2-nulled mutant mice show reduced levels of Alpha, Mu and Pi GSTs, as well as reduced expression of *MAPEG* genes [[110], [111], [112]]. In plants, during biotic (pathogens) and abiotic stress (cold, drought, heavy metals, high salt), the induction of GST expression has been reported [[113], [114], [115], [116], [117], [118]]. These studies indicate the importance of the different types of GSTs during antioxidant response.

## 3 BSH and bacillithiol transferases

### 3.1 Bacillithiol and its cellular functions

Most low G + C Gram-positive bacteria (e.g. *Bacilli,* some *Staphylococci*, and *Streptococci*) utilize bacillithiol (BSH; α-anomeric glycoside of L-cysteinyl-d-glucosamine with l-malate) as their major LMW thiol. BSH is also detected in a few Gram-negative bacteria, including *Bacteroidetes* and *Acidobacteria* [[119], [120], [121], [122]]. Similar to GSH, BSH protects proteins from overoxidation through bacillithiolation, a mixed disulfide bond formed between a protein and BSH (PS-SB). Bacilliredoxins A and B (BrxA and BrxB) reduce this mixed disulfide bond, resulting in the release of the reduced form of the protein and a bacillithiolated Brx (BrxS-SB). Another molecule of BSH reduces this second mixed disulfide bond, where bacillithiol disulfide (BSSB) is formed. The NADPH-dependent bacillithiol disulfide reductase (Bdr, also known as YpdA) reduces BSSB into two molecules of BSH [24,[123], [124], [125], [126]]. A new bacilliredoxin, BrxC, which can reduce the BSH and BrxB mixed disulfide bond (BrxB-SB) and the BSH and Bdr mixed disulfide bond (BdrS-SB) was identified [127].

**Fig. 3:**
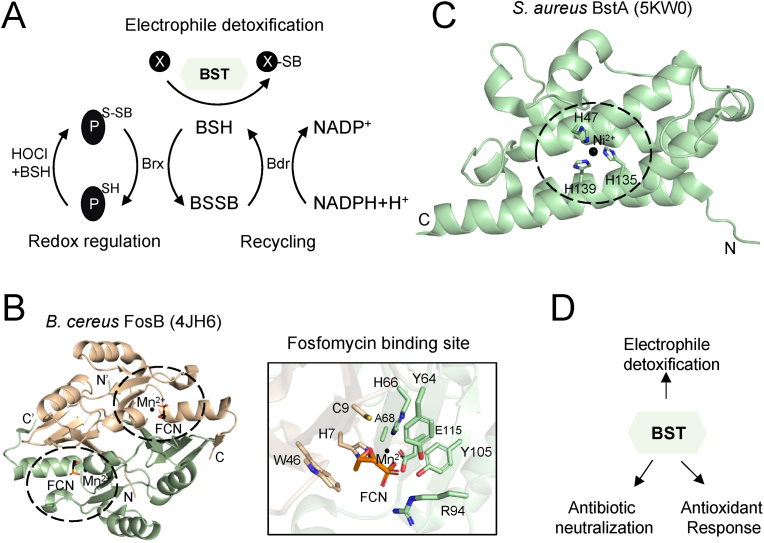
Structural architecture and cellular functions of bacillithiol transferases. **(A)** BSH participates in diverse cellular functions including electrophile (X) detoxification, which is catalyzed by BstA and FosB bacillithiol transferases. BSH also protects proteins from overoxidation through the formation of a mixed disulfide bond with protein thiols, termed protein bacillithiolation. Brx enzymes reduce the bacillithiolated proteins and use the BSH/Bdr pathway to restore their active form. **(B)** The homodimeric *B. cereus* FosB structure (PDB: 4JH6) is shown. Monomer 1 is shown in wheat color, and monomer 2, in light green. Each monomer contains Mn^2+^ (black sphere) and fosfomycin (FCN – black sticks). The metal and fosfomycin binding sites are shown in black circles. The insert shows the fosfomycin binding site residues, fosfomycin and Mn^2+^. **(C)** The *S. aureus* BstA structure (PDB: 5KW0) is shown in light green. The Ni^2+^ ion is shown in black sphere and the metal binding site is shown in black circle. The His residues stabilizing the Ni^2+^ ion are shown in green sticks. The N-terminus (N) and C-terminus (C) are indicated. **(D)** Bacillithiol transferases participate in diverse cellular functions, including electrophile detoxification, antioxidant response and antibiotic neutralization. (For interpretation of the references to color in this figure legend, the reader is referred to the Web version of this article.)

In addition to protecting proteins from oxidative damage through bacillithiolation, BSH participates in diverse cellular functions including detoxification of electrophiles, alkylating agents, and toxic metals, as well as contributing to the cellular metal and sulfide homeostasis [120,[128], [129], [130]] (Fig. 3A). For instance, methylglyoxal, a reactive toxic by-product of glycolysis can be detoxified by BSH in *B. subtilis*. The cellular functions of bacillithiol transferases are discussed in the following sections.

### 3.2 Bacillithiol transferase superfamilies and classes

Two types of bacillithiol transferases have been reported in Firmicutes: a) FosB – a member of the VOC superfamily and b) bacillithiol transferases (BST) from the *S*-transferase like (STL) superfamily (previously known as the DinB/YfiT-like putative metalloenzymes). The two types of BSH transferases share no sequence and structural similarities. The VOC superfamily includes metal-dependent fosfomycin resistance enzymes, e.g. FosA, FosB and FosX. FosA and FosB catalyse the conjugation of GSH and BSH/l-cysteine (L-Cys) onto C1 carbon of the fosfomycin oxirane ring, respectively, while FosX catalyzes the hydration of fosfomycin [10,53,[131], [132], [133]]. All conjugated products lack bactericidal properties. A single *fosB* gene is present in bacteria [132]. The metal dependent BSH transferase, BstA, belongs to the STL superfamily, which includes mycothiol transferase (MST) enzymes. BstA catalyzes the conjugation of BSH onto electrophiles, which follow the cellular detoxification path. The expression of BST varies in species, with only one BST (BstA) present in *S. aureus*, and 8 BSTs (BstA-H) reported in *Bacillus subtilis* that have varying levels of BSH transferase activities [11,132,134].

### 3.3 Structural architecture of bacillithiol transferases

The homodimeric FosB structure possesses domain-swapped arrangement of tandem βαβββ motifs (PDB: 4JH6), similar to other VOC superfamily members, FosA and FosX [133,135]. Within the FosB U-shaped active site, a metal ion is coordinated by histidine and glutamate residues. The metal ion, along with the conserved residues within the active site, anchor fosfomycin in a predisposed position where its C1 carbon (oxirane ring) faces towards the solvent accessible surface [133,135] (Fig. 3B – black circles). This could facilitate the nucleophilic attack by the thiol group of BSH onto the fosfomycin C1. Although the fosfomycin binding site is reported within the FosB active site, the binding mode and stabilization interactions of BSH to the fosfomycin-bound FosB structure remain to be further investigated. The structure of FosB in complex with BSH was determined, where only the Cys moiety of BSH was observed due to the high flexibility of other parts of the molecule structure. BSH thiol group was covalently bound to FosB Cys9 (PDB: 4NB0). It is speculated that the disulfide bond formed between BSH and Cys9 could be due to crystallization conditions.

As a member of the STL superfamily, the homodimeric *S. aureus* BstA has a typical DinB domain comprised of a four-helix bundle (PDB: 5KW0) [11,136]. It is a metalloenzyme with a metal ion stabilized by conserved His residues (Fig. 3C – black circle) [136,137]. A possible dimeric interface involves residues of helices 1 and 4 of each monomer. The reported putative BstA active site pocket is composed of helices 2 and 4 of the four-helix bundle together with the three loops which extend out of the helices (Fig. 3C). Although a nickel ion was observed within the structure (due to purification conditions), further studies demonstrated that in the presence of zinc ion, BstA has a higher BSH transferase activity [136]. Within the putative active site pocket, polar and positively charged residues are present, which may stabilize the negatively charged malate moiety of bacillithiol. On the other side of the metal ion, non-polar residues are present, which may participate in the stabilization of electrophilic substrates [136]. Future studies of BSH-bound and/or electrophilic substrate-bound BstA structure(s) could reveal the detailed BSH and electrophilic substrate binding sites and stabilization interactions. The structure of BSH transferase YfiT from *B. subtilis* (PDB: 1RXQ) is reported, which also contains the four-helix bundle with a metal ion stabilized by three His residues. Unlike *S. aureus* BstA, YfiT forms a dimer interface with helix 2 and 3, and the loops connecting the helices are significantly different [136,137]. The differences between the active sites of both enzymes suggest that they may bind BSH and substrates differently.

### 3.4 Function and regulation of bacillithiol transferases

FosB utilizes BSH for fosfomycin detoxification and confers antibiotic resistance in several human pathogens including *S. aureus* and *B. anthracis* [132]. Fosfomycin is a widely used antibiotic that covalently modifies the active site cysteine residue of MurA (UDP-N-acetylglucosamine enolpyruvyl transferase) which is essential for bacterial cell wall synthesis [128]. *S. aureus fosB* defective mutants show increased sensitivity towards fosfomycin, diamide and H_2_O_2_ treatments [132,138]. The level of both BSH and NADPH decreases in the defective *fosB* mutants, suggesting that FosB may have a role in the oxidative stress response [138,139]. In naturally occurring methicillin-resistant (MRSA) *S. aureus*, *fosB* gene is upregulated in some clonal lineages, suggesting the development of fosfomycin resistance [140].

*S. aureus* and *B. subtilis* BstA enzymes catalyse the conjugation of BSH onto a variety of electrophilic substrates including cerulenin, CDNB, cumene hydroperoxide, chlorinated hydrocarbons and monobromobimane (Fig. 3D) [12,125,141]. However, physiological substrates of BstA/YfiT remain to be investigated. Upon BstA-catalyzed conjugation of BSH to electrophiles, bacillithiol-electrophile conjugates (BSRs) are formed. BSH *S*-conjugate amidase (Bca) or BSH biosynthesis deacetylase (BshB2) cleaves the BSR into GlcNAc-Mal (N-acetylglucosamine malate) and mercapturic acid (CysSR) [12,125,137]. The latter is exported out of the cell by the potential efflux pumps (encoded by yfiS and yfiU genes) [12,125]. BSH also contributes to the pathogenicity and antibiotic resistance mechanisms of Firmicutes through the detoxification of antibiotics by means of bacillithiol transferases.

The expression of BST is regulated in response to antibacterial treatment, organic electrophiles or oxidising agents. Addition of salicylic acid or mammalian peptidoglycan recognition proteins causes increased expression of BstB in *B. subtilis* [132,142]. Antibacterial treatment with methylhydroquinone also increases the expression of BstB and BstD in *B. subtilis* [132,134]. Similarly, the expression of *S. aureus* BstA increases in response to treatment with H_2_O_2_, hypochlorite (HOCl) and azurophilic granule proteins produced by neutrophils [141].

Overall, BSH transferases FosB and BstA play an important role in neutralizing antibiotics and detoxifying electrophiles (Fig. 3D), however, their potential role in the antioxidant defense mechanisms, such as catalyzing the bacillithiolation of proteins, similar to some GST members, remains to be determined.

## 4 MSH and mycothiol transferases

### 4.1 MSH and its cellular functions

**Fig. 4:**
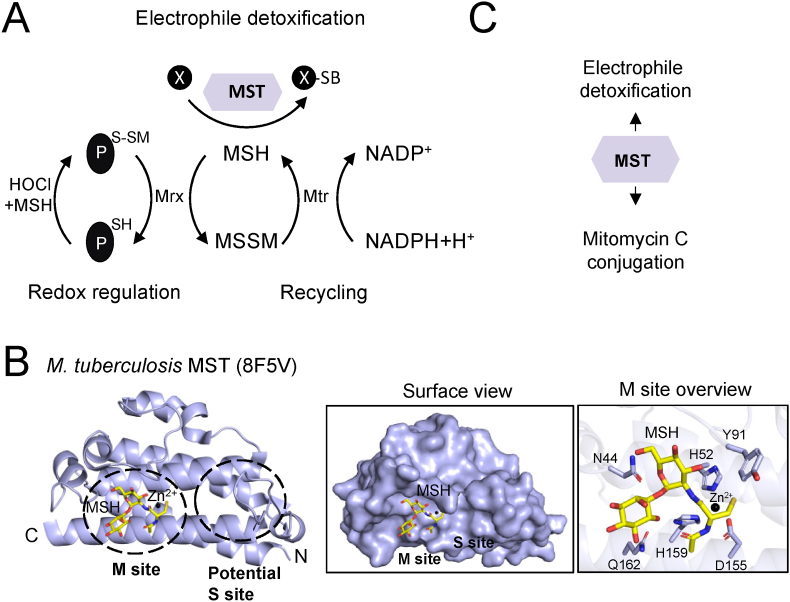
Structural architecture and cellular functions of mycothiol transferase. **(A)** MSH participates in diverse cellular functions including electrophile (X) detoxification, which is catalysed by MST. MSH also protects proteins from overoxidation through the formation of a mixed disulfide bond with protein thiols, termed protein mycothiolation. Mrx reduces the mycothiolated proteins and uses the MSH/Mtr pathway to restore its active form. **(B)** The *M. tuberculosis* MST structure (PDB: 8F5V) is shown in purple. The structure contains a metal (Zn^2+^ - black sphere) and a mycothiol molecule (MSH – yellow sticks). The MSH binding site (M site) and the potential substrate binding site (potential S site) are indicated. The N-terminus (N) and C-terminus (C) are also indicated. The left insert shows a surface view of the MST M site and the potential S site. The right insert shows the residues (purple) that stabilize MSH and Zn^2+^ ion within the M site. **(C)** The mycothiol transferase mainly participates in electrophile and mitomycin C detoxification, amongst other cellular functions. (For interpretation of the references to color in this figure legend, the reader is referred to the Web version of this article.)

Mycothiol (MSH; AcCys-GlcN-Ins; 1-D-myo-inositol 2 (N-acetyl-L-cysteinyl)amido-2-deoxy-α-d-glucopyranoside) is the major LMW thiol in *Actinomycetes* (e.g. *Mycobacterium tuberculosis*, *Mycobacterium smegmatis*, and *Corynebacterium diphtheriae*) [[143], [144], [145]]. It is involved in diverse cellular processes, including redox signalling and regulation, xenobiotic detoxification, antibiotic resistance and prodrug activation, among others [21,125,[145], [146], [147], [148], [149], [150]]. Upon cellular oxidative stress, MSH protects proteins from oxidative damage through protein mycothiolation, where a mixed disulfide bond is formed between MSH and the protein cysteine thiol (PS-SM). Mycoredoxin 1 (Mrx1), a glutaredoxin homologue, catalyzes the reduction of the PS-SM disulfide bond, resulting in the transfer of MSH from the modified protein onto Mrx1 (MrxS-SM). The mycothiolated Mrx1 (MrxS-SM) is reduced by another molecule of MSH, leading to the formation of mycothiol disulfide (MSSM). MSSM is further reduced to two MSH molecules by the NADPH-dependent mycothiol disulfide reductase (Mtr) (Fig. 4A) [145,147,151]. This regenerates the pool of reduced MSH and maintains the reduced cellular environment during ROS detoxification. MSH also regulates the function of proteins under non-stressed conditions. For example, upon substrate reduction, *C. diphtheriae* methionine sulfoxide reductase A (MsrA) forms a sulfenic acid on its catalytic cysteine residue, which becomes mycothiolated and uses the MSH/Mrx1/Mtr pathway to recycle MsrA and restore its active form [152]. Examples of proteins that are regulated by MSH include thiol peroxidase (Tpx), alkyl peroxide reductase E (AhpE), MsrA, MsrB, mycothiol peroxidase (Mpx), GAPDH and the organic hydroperoxides stress regulator (OhsR) [21,[152], [153], [154], [155], [156]].

MSH participates in the detoxification of antibiotics and xenobiotics (Fig. 4A) [145,149]. During arsenate detoxification, the arsenate reductases (ArsC1/ArsC2) catalyse the conjugation of MSH and arsenate (As(V)) to form MSH-conjugated arsenate (As(V)-SM), which is subsequently reduced by Mrx1 to arsenite (As(III)) [21,157]. Mycothiol can spontaneously conjugate to nitric oxide and formaldehyde, forming *S*-nitromycothiolates (MSNO) and *S*-hydroxymethyl-MSH, respectively. With its dual function, *S*-nitrosomycothiol reductase/-formaldehyde dehydrogenase (MscR) detoxifies both compounds. Additionally, *M*. *Smegmatis* mutants lacking MSH biosynthesis or MscR had decreased biofilm formation [125,148,149,158]. Recent studies also indicate the involvement of S-formyl-mycothiol hydrolase (Fmh) in the regeneration of MSH during the detoxification of formaldehyde [159]. Mycothiol transferases (MST) can catalyse the conjugation of MSH onto electrophiles (RX), leading to the generation of MS-electrophile. This conjugate is further hydrolysed by the mycothiol *S*-conjugated amidase (Mca) to glucosaminylinositol (GlcN-Ins), which is recycled for MSH biosynthesis, and mercapturic acid derivative (AcCysR), which is exported out of the cell [12,160]. The cellular functions of MSH transferases are discussed in the following section.

### 4.2 Structural architecture and cellular functions of MST

MST is a member of the DinB (DNA damage inducible gene) superfamily, originally identified in *M. smegmatis* and *M. tuberculosis*. MST from *M. tuberculosis* is a homodimeric metalloprotein, which has the typical DinB domain composed of a four-helix bundle [161]. The homodimeric structure of MST contains one metal ion and one molecule of MSH per monomer (Fig. 4B). The MST metal-binding site includes helices 2 and 8 (from the four-helix bundle), which contain the metal-binding residues (two His and one Asp) [161]. This differs from other DinB superfamily members that stabilize the metal via three conserved His residues [162]. MSH is stabilized through interactions with the metal, Asn, Asp and the backbone of Gly and Gln. Structural comparison between the MSH/metal bound and ligand unbound structures suggests a possible second substrate binding site located close to the MSH binding site. Within the second potential substrate binding site, there are modifications in the dynamics of loop 4 and rotamer changes in Trp and Tyr residues located near the catalytic metal [161]. The study suggests that these modifications influence the electrostatic surface charge of this site to a more neutral and less positively charged environment compared to the metal binding site. This could allow the accommodation of a molecule that has both hydrophobic and hydrophilic properties [161]. Further biochemical and structural studies could shed light on the nature of the MST substrates, their binding sites and the catalytic mechanism.

The cellular functions of MSTs remain largely elusive compared to GST due to the limited studies on these proteins. MSTs are shown to catalyse the transfer of MSH to monochlorobimane (mBCl), 1-chloro-2,4-dinitrobenzene (CDNB) and mitomycin C (chemotherapeutic drug) (Fig. 4C) [12]. Further studies are required to identify MST substrates and define the biological role of these enzymes. The identity of a second type of mycothiol transferase has been recently reported, which belongs to the Xi class of transferases (also includes GSTs). Interestingly, MST Xi was found to possess mycoredoxin and dehydroascorbate reductase activities but does not have mycothiol transferase activity [163]. The redox regulation by MSTs has not been extensively studied, however, MST (DinB superfamily) and MST Xi are reported to be mycothiolated on Cys36 (co-crystallized 3D structure) and the catalytic Cys67 (in the presence of H_2_O_2_; uses MSH/Mtr pathway for recycling), respectively [161,163]. Further studies on the catalytic function of MSTs during cellular stress could enhance the knowledge on how these enzymes are regulated.

## 5 T(SH)_2_ and trypanothione transferases

Trypanothione (N^1^,N^8^-bis(glutathionyl)spermidine - T(SH)_2_) is composed of bis-glutathione molecules joined by a spermidine linker. In trypanosomatids (protozoan parasites e.g. trypanosomes and leishmania) it is the major LMW thiol, which plays a role in the cellular defence against oxidative stress and certain heavy metals (e.g. cadmium ion (Cd^2+^) or mercury (Hg^+^)) [22,[164], [165], [166], [167]]. During oxidative stress, T(SH)_2_ protects proteins from irreversible overoxidation through trypanothionylation, a mixed disulfide bond formed between the protein thiol and T(SH)_2_ [22]. More research is necessary to elucidate the mechanism of trypanothione-mediated protein regulation. In addition, T(SH)_2_ is indirectly involved in hydroperoxide scavenging. The trypanoredoxin peroxidase reduces H_2_O_2_ and organic hydroperoxides generated during oxidative stress. Tryparedoxin then reduces the oxidized trypanoredoxin peroxidase and uses the T(SH)_2_ and the NADPH-dependent trypanothione reductase as a recycling system [164,168]. Consequently, multiple trypanothione-dependent enzymes have been the subject of drug-target studies [169].

Trypanothione transferase (TST) activity was detected in lysates of trypanosomatids (e.g. *Crithidia fasciculata*). The activity of endogenous *C. fasciculata* TST was purified and identified as the ribosomal elongation factor 1B complex (eEF1B). The eEF1B contains three subunits (α, β, and γ) of which the γ subunit was established to possess the TST activity. Only in the presence of all three subunits, eEF1B exhibits the TST activity. Among a range of substrates, *C. fasciculata* TST was shown to catalyse the conjugation of T(SH)_2_ onto CDNB; however*, Leishmenia major* TST was shown to catalyse T(SH)_2_ conjugation to hydrophobic hydroperoxides (e.g. 4-hydroxy-2-nonenal and thymine hydroperoxide) [9,170]. Interestingly, TST is not involved in the resistance and detoxification of antimonial drugs in *Leishmania tarentolae* [171]. Other physiological functions of TST have yet to be identified to establish its biological role and determine whether TST would be a good drug target.

## 6 Redox regulation by ergothioneine

Ergothioneine is an unusual, naturally occurring LMW thiol that predominately exists as thione under physiological conditions. The lower reactivity and a very high redox potential (Table 1) make it highly resistant to autoxidation. ET also shows better chemical and thermal stability when compared to other LMW thiols [172]. ET is produced in large quantities by some bacteria and fungi, but the molecular pathway for its biosynthesis does not exist in animals and humans [173]. They acquire ET from the diet and the transport is facilitated through a membrane transporter SLC22A4 (OCTN1) which has wide distribution in many cells and tissues [174]. The knockout of the SLC22A4 transporter in model organisms is not lethal but increases susceptibility to oxidative stress and inflammation [175,176]. These findings are in agreement with the antioxidant and cytoprotective function of ET. Extensive studies from various laboratories indicate that ET is an effective scavenger of singlet oxygen, superoxide, hydroxyl radical, H_2_O_2_, lipid peroxides and nitric oxide derivatives [177,178]. ET has been also reported to inhibit the oxidative damage to mtDNA [179]. The reduction of oxidized ET can involve ascorbate, glutathione reductase in presence of GSH, or thioredoxin reductase [180,181]. The activation of a master regulator of antioxidative responses, transcription factor NRF2 (nuclear factor erythroid 2-related factor 2) by ET has been demonstrated in several studies [182,183], but the molecular mechanism remains to be investigated.

Blood levels of ET was found to decline with age and the decrease has been associated with cardiovascular diseases, neurodegeneration and mild cognitive impairment [[184], [185], [186]]. Sufficient preclinical and epidemiological data are available to justify the use of ET for the prevention of cardiovascular diseases, neurodegeneration, type 2 diabetes, and the promotion of healthy ageing. The broader application of ET for use in general health and wellbeing has been facilitated by its approval in the EU and USA as a safe supplement in human nutrition, cosmetics and pharmaceutical products.

The existence of enzymes which function as transferases for other LMW thiols in promoting the detoxification of various xenobiotics and preventing irreversible overoxidation of cysteine residues under oxidative stress has prompted researchers to identify such enzyme(s) for ET. In one study, ET was found to react with CDNB *in vitro* and *in vivo* [187]. Moreover, the toxicity of CDNB was shown to be significantly decreased in NCI–H441 cells co-incubated with ET. Further studies are required to investigate and identify the enzyme(s), involved in the ET sulfur transferase activity.

## 7 Redox regulation by coenzyme A

CoA is an essential cofactor for the viability of all living cells. In contrast to other LMW thiols, CoA contains the thiol group at the tip of a long and flexible pantetheine tail which is attached to the ADP moiety. This permits CoA to be involved in diverse biochemical reactions and the formation of thioester bonds with “energy-rich” carboxylic acids. The research on CoA and its thioester derivatives has been focused for many years on studying their function in cellular metabolism, signal transduction and the regulation of gene expression [188,189]. Abnormal biosynthesis and homeostasis of CoA and its thioester derivatives have been associated with various human pathologies, including neurodegeneration, cancer and metabolic disorders.

Studying the role of CoA in redox regulation has been hampered by the lack of reagents and methodologies. The development of highly specific anti-CoA monoclonal antibodies and a reliable MS-based methodology was instrumental for the identification and characterisation of protein thiolation by CoA (termed CoAlation), as a widespread and reversible PTM in redox regulation in both eukaryotic and prokaryotic cells [23,101,[190], [191], [192], [193], [194], [195]]. These advances have allowed the identification of more than two thousand CoAlated proteins in mammalian cells/tissues and bacteria [50,52]. CoAlation was shown to modify the activity, subcellular localization and the conformation of modified proteins, and to protect them from irreversible overoxidation [192,[196], [197], [198], [199], [200]]. The identity of enzymes which are involved in redox regulation by CoA and the CoAlation/deCoAlation cycle is the focus on ongoing research. By analogy to protein glutathionylation, the existence of dedicated enzymes facilitating the antioxidant function of CoA is anticipated. These include: a) CoA *S*-transferase (a member of the CoA transferases), to promote the forward reaction of CoAlation; b) CoAredoxins, to mediate deCoAlation of modified proteins/molecules [201]; c) CoA disulfide reductase (CoADR), to reduce CoA disulfide (CoASSCoA) which is generated during oxidative stress [202]; and d) CoA-dependent peroxidase(s), to reduce H_2_O_2_ and organic hydroperoxides. Protein CoAlation could occur via the disulfide exchange mechanism (experimentally demonstrated) or enzymatic conjugation of CoA to protein cysteine thiols mediated by a currently unknown transferase (analogous to GST Pi). Considering that the pKa value of the CoA thiol group is high (∼9.8) (Table 1), when compared to GSH, BSH and MSH, the existence of a dedicated enzyme for promoting efficient protein CoAlation is plausible. Moreover, CoA *S*-transferase(s) can be responsible for protecting cells against oxidative stress by-products and the toxic impacts of xenobiotics.

## 8 Future perspectives

Significant advances have been made in understanding the regulation and function of GSTs, and the development of research tools and resources targeting these enzymes. Thousands of papers have been published on GSTs since their discovery in seventies of the last century. These studies paved the way for the identification and functional characterisation of LMW thiol transferases for MSH, BSH and T(SH)_2_. These enzymes are highly divergent in their structure, function and expression pattern, as they need to cope with an equally diverse array of endogenous or exogenous toxic metabolites and ROS. New advances are expected over the next decade in the development of research reagents and methodologies, which can promote our understanding of critical roles of recently identified BSTs, MST and TST in drug and xenobiotic metabolism, redox regulation, and antioxidant defence, especially in pathogenic microorganisms.

Although ET was isolated a century ago, the molecular mechanisms of its antioxidant and cytoprotective function has started to emerge in the beginning of this century with the identification and genetic analysis of the ET transporter. Recent advances in demonstrating specific interaction between ET and CDNB *in vitro* and in cells will pave the way for studying non-enzymatic and enzymatic mechanisms of xenobiotic deactivation by this LMW thiol.

With many questions about the antioxidant function of CoA outstanding, it is a fascinating time for molecular dissection of the CoAlation/deCoAlation cycle and studying the role of CoA in pathologies associated with oxidative stress, such as neurodegeneration and cancer. The identification and characterisation of the enzyme(s) which catalyse the transfer of CoA to electrophilic compounds or cysteine residues modified by oxidative stress is expected in the next few years. Within the superfamily of transferases, there are several classes of enzymes which catalyse the transfer a functional CoA group to various substrates. Class I CoA transferases catalyse the transfer of CoA group from an acyl-CoA donor to free carboxylic acid. Members of class II catalyse the conjugation of CoA to free fatty acids or antibiotics. Class III CoA transferases use acyl-carrier protein as the substrate. Nomenclature wise, these enzymes are not often referred as 'CoA transferases', but named as Acyl-CoA synthetases and CoA ligases. The nomenclature and substrate specificity of CoA transferases has to be reviewed taking into account their involvement in cellular metabolic processes or redox regulation.

## Submission declaration and verification

This work has not been published previously and is not under consideration for publication elsewhere. Moreover, both authors fully approve of the contents of this report.

## CRediT authorship contribution statement

**Maria-Armineh Tossounian:** Writing – review & editing, Writing – original draft. **Yuhan Zhao:** Writing – review & editing, Writing – original draft. **Bess Yi Kun Yu:** Writing – review & editing, Writing – original draft. **Samuel A. Markey:** Writing – review & editing, Writing – original draft. **Oksana Malanchuk:** Writing – review & editing, Writing – original draft. **Yuejia Zhu:** Writing – review & editing, Writing – original draft. **Amanda Cain:** Writing – review & editing. **Ivan Gout:** Writing – review & editing, Writing – original draft, Supervision, Funding acquisition, Conceptualization.

## Declaration of competing interest

The authors do not have a conflict of interest to report.

## Data Availability

Data will be made available on request.
